# Current trends on immunotherapy for oncology

**DOI:** 10.6026/973206300211880

**Published:** 2025-07-31

**Authors:** Neya Kavya Chander, Prashannalakshmi A., Phanish Chandra Ravi, Aravind Muthiah, Yagvalkya Sharma, Gajalakshmi Suriyanarayanan, Shakthipriya P.S

**Affiliations:** 1 Institute of Internal Medicine, Madras Medical College & Rajiv Gandhi Government General Hospital, Chennai, India; 2 Department of Medicine, Tashkent Medical Academy, Urgench branch, Uzbekistan; 3 Department of Internal Medicine, SVS Medical College, Mahabubnagar, India; 4 Department of Medicine, Tirunelveli Medical College, Tirunelveli, India; 5 Department of Research, Kalp Research Work, Mathura, Uttar Pradesh, India; 6 Department of Community Medicine, Veer Chandra Singh Garhwali Government Institute of Medical Science and Research, Srinagar, Pauri Garhwal, Uttarakhand, India; 7 Institute of Internal Medicine, Madras Medical College, Chennai, India

**Keywords:** Cancer immunotherapy, immune checkpoint inhibitors, CAR T-cell therapy, tumor microenvironment, immune-related adverse events, precision oncology, combination therapy

## Abstract

Immunotherapy has revolutionized oncology by harnessing the patient's own immune system to recognize and eliminate tumor cells, leading to durable responses in a variety of malignancies. Over the past decade, immune checkpoint inhibitors targeting CTLA-4, PD-1, and PD-L1 have become standard of care across multiple solid tumors, while chimeric antigen receptor (CAR) T-cell therapies have demonstrated remarkable efficacy in hematologic cancers. Despite these successes, challenges such as primary and acquired resistance, immune-related toxicities, and high treatment costs continue to limit broader application. Emerging strategies including novel checkpoint targets (*e.g.*, LAG-3, TIGIT), next-generation cellular therapies, cancer vaccines, and oncolytic viruses are under active investigation to expand therapeutic options and improve patient outcomes. Biomarker development for patient selection and combination regimens with chemotherapy, targeted agents, and radiation are critical to overcoming resistance mechanisms. Looking ahead, personalized immunotherapy approaches leveraging tumor neoantigens and the tumor microenvironment hold promise for more precise and effective treatments.

## Background:

Cancer immunotherapy has fundamentally redefined the oncologic treatment paradigm by leveraging the adaptive and innate arms of the immune system to recognize and eradicate malignant cells with high specificity. The concept of immunosurveillance wherein cytotoxic T lymphocytes (CTLs), natural killer (NK) cells, and antigen-presenting dendritic cells continuously patrol for nascent tumor neoantigens has evolved into the more nuanced model of immunoediting [1]. In this framework, the interplay between tumor immunogenicity and host immunity proceeds through phases of elimination (immune-mediated tumor cell clearance), equilibrium (a dynamic balance between immune pressure and tumor adaptation), and escape (tumor evolution to evade immune detection) [2]. Molecular dissection of inhibitory pathways, such as CTLA-4/CD28 and PD-1/PD-L1 checkpoints, revealed critical "brakes" on T-cell activation that tumors co-opt to create an immunosuppressive microenvironment. Monoclonal antibodies targeting these checkpoints have demonstrated durable antitumor responses by restoring effector T-cell function and facilitating epitope spreading [3]. Concurrently, adoptive cell transfer approaches most prominently chimeric antigen receptor (CAR) T-cell technology have showcased the therapeutic potential of genetically reprogramming patient T cells to recognize lineage-specific antigens with high affinity [4]. These engineered lymphocytes mediate immediate cytotoxicity through perforin/granzyme release and sustained immune surveillance via cytokine secretion. Beyond cellular therapies, oncolytic viruses and neoantigen-based vaccines are being engineered to prime endogenous immune responses and reshape tumor-infiltrating lymphocyte repertoires [5]. Despite these advances, significant hurdles remain: heterogeneity in tumor mutational burden leads to variable neoantigen landscapes and limits broad efficacy; the development of immune-related adverse events necessitates vigilant toxicity management; and both intrinsic and acquired resistance mechanisms such as upregulation of alternative checkpoint receptors or stromal barrier formation undermine long-term control [6]. This comprehensive review will synthesize the mechanistic underpinnings of current immunotherapeutic modalities, evaluate strategies for biomarker-driven patient stratification, and highlight emerging innovations aimed at overcoming resistance and extending the benefits of immune-based oncology.

## Literature search strategy:

A comprehensive literature search was performed to identify studies, reviews, and clinical reports relevant to contemporary immunotherapeutic approaches in oncology. Three primary electronic databases PubMed/MEDLINE, Scopus, and Web of Science were queried covering publications from January 2015 through June 2025. Search terms combined controlled vocabulary and free-text terms, including "cancer immunotherapy," "immune checkpoint inhibitor," "CAR T-cell," "cancer vaccine," "oncolytic virus," "immune-related adverse event," and "tumor microenvironment," using Boolean operators (*e.g.*, "immunotherapy AND oncology," "PD-1 OR PD-L1"). Although this review does not adhere to formal systematic-review methodology, the approach was designed to ensure transparency and reproducibility in study identification and selection, thereby providing a representative overview of current trends and innovations in cancer immunotherapy.

## Overview of mechanisms of action:

## Immune checkpoint blockade:

Immune checkpoint inhibitors function by disrupting inhibitory receptor-ligand interactions that dampen T-cell activation. CTLA-4 blockade with monoclonal antibodies interferes early in the priming phase within lymphoid organs, preventing CTLA-4 from outcompeting CD28 for B7 co-stimulatory ligands on antigen-presenting cells. In contrast, PD-1/PD-L1 inhibitors act predominantly in peripheral tissues and within the tumor microenvironment, reversing T-cell exhaustion by blocking PD-1 on effector T cells or PD-L1 on tumor and stromal cells [7]. The net effect is restoration of T-cell proliferation, cytokine production, and cytolytic function, leading to enhanced recognition and destruction of tumor cells.

## Adoptive cell therapies:

Adoptive transfer approaches involve the ex vivo manipulation of a patient's lymphocytes to enhance tumor specificity and effector function before reinfusion. Chimeric antigen receptor (CAR) T cells are engineered to express synthetic receptors comprising an extracellular single-chain variable fragment (scFv) that binds a tumor-associated antigen, fused to intracellular signaling domains (*e.g.*, CD3ζ and 4-1BB or CD28). Upon antigen engagement, CAR T cells undergo rapid activation, release perforin and granzyme, and proliferate *in vivo*, mediating potent cytotoxicity. T-cell receptor (TCR)-engineered therapies utilize high-affinity TCRs targeting intracellular antigens presented on MHC, allowing recognition of a broader antigen repertoire but requiring HLA matching. Both modalities rely on lymphodepleting conditioning regimens to create "space" and homeostatic cytokine milieus that enhance engraftment and persistence [8].

## Cancer vaccines and oncolytic viruses:

Therapeutic cancer vaccines aim to induce or amplify tumor-specific immune responses by delivering tumor antigens in the form of peptides, proteins, nucleic acids, or dendritic cell formulations. These vaccines promote antigen uptake by dendritic cells, cross-presentation to CD8+ T cells, and generation of memory responses. Neoantigen-based vaccines, customized to patient-specific tumor mutations, have demonstrated the ability to elicit polyclonal, high-avidity T-cell populations. Oncolytic viruses, such as genetically attenuated herpesviruses or adenoviruses, selectively replicate in tumor cells, causing immunogenic cell death and release of tumor antigens in an inflammatory context, thereby acting as in situ vaccines and remodeling the immunosuppressive microenvironment [9].

## Cytokine therapies:

Recombinant cytokines-most notably interleukin-2 (IL-2) and interferon-α (IFN-α)-provide non-specific immune stimulation by enhancing proliferation and activation of T cells and natural killer cells. High-dose IL-2 can induce durable remissions in metastatic melanoma and renal cell carcinoma but is limited by severe capillary leak and organ toxicities. Modified cytokine constructs and engineered cytokine-antibody fusion proteins (immunocytokines) are under development to improve therapeutic index by targeting cytokine activity to the tumor site or by attenuating systemic exposure [10].

## Current clinical applications and recent approvals:

## Checkpoint inhibitors in solid tumors:

Immune checkpoint blockade has established new standards of care for multiple solid malignancies. PD-1 inhibitors are now routinely administered in advanced melanoma, non-small cell lung cancer (NSCLC), renal cell carcinoma, and head and neck squamous cell carcinoma, either as monotherapy or in combination with chemotherapy. PD-L1 inhibitors have similarly gained approval for urothelial carcinoma and gastric cancer, where durable responses have translated into meaningful overall survival benefits. Dual blockade of CTLA-4 and PD-1 has further improved response rates in high-mutational burden tumors, although at the expense of increased immune-related toxicity [11].

## CAR-T cell therapies in hematologic malignancies:

Adoptive transfer of CD19-targeted CAR-T cells has revolutionised treatment of relapsed/refractory B-cell acute lymphoblastic leukemia (ALL) and diffuse large B-cell lymphoma (DLBCL). Following lymphodepleting chemotherapy, reinfused CAR-T cells expand dramatically *in vivo*, eradicating minimal residual disease in a substantial proportion of patients. Subsequent approvals have extended CAR-T indications to mantle cell lymphoma and multiple myeloma (via BCMA-directed constructs). Real-world data demonstrate high complete remission rates, although late relapses underscore the need for strategies to prolong CAR-T cell persistence and mitigate antigen-negative escape [12].

## Emerging vaccine and oncolytic platforms:

Cancer vaccines have recently achieved milestones with approval of an mRNA-based neoantigen vaccine in adjuvant melanoma, demonstrating favorable safety and immunogenicity profiles. Similarly, oncolytic virus therapy has been approved for melanoma with intracerebral metastases, leveraging direct oncolysis and systemic immune activation. Early-phase trials of peptide-based and dendritic-cell vaccines are ongoing in glioblastoma and pancreatic cancer, where conventional immunotherapies have historically shown limited efficacy [13].

## Bispecific and next-generation antibody constructs:

Beyond monospecific antibodies, bispecific T-cell engagers (BiTEs) that simultaneously bind CD3 on T cells and tumor antigens have received approval in acute lymphoblastic leukemia and are under investigation in non-Hodgkin lymphomas. Additionally, Fc-optimized and checkpoint-bispecific antibodies are in clinical development, designed to recruit both innate and adaptive effector mechanisms while minimizing systemic toxicity. These approvals underscore a rapidly expand therapeutic landscape in which immunotherapy is integrated across tumor types and disease stages transforming previously intractable cancers into manageable conditions for a growing subset of patients [14].

## Biomarkers for response and resistance:

The identification and validation of predictive biomarkers are pivotal for optimizing patient selection and improving therapeutic outcomes in cancer immunotherapy. Tumor PD-L1 expression, quantified by immunohistochemistry on tumor cells or infiltrating immune cells, serves as a provisional companion diagnostic for PD-1/PD-L1 inhibitors; however, assay variability and dynamic regulation of PD-L1 limit its predictive precision. Tumor mutational burden (TMB), reflecting the total number of somatic mutations per megabase, correlates with neoantigen load and has been associated with higher response rates to checkpoint blockade, particularly in melanoma and NSCLC [15]. Despite this, TMB thresholds lack standardization across sequencing platforms and do not account for antigen presentation efficiency or immune contexture. Gene expression signatures such as the interferon-γ-related mRNA profile provide insight into preexisting T-cell inflammation within the tumor microenvironment and may predict benefit from PD-1 pathway inhibition. In addition, circulating biomarkers, including peripheral blood T-cell receptor (TCR) clonality and early changes in circulating tumor DNA (ctDNA), are under investigation as minimally invasive surrogates for tumor response and emerging resistance. Mechanisms of resistance encompass loss of antigen presentation through β2-microglobulin mutations, upregulation of alternate inhibitory receptors (*e.g.*, TIM-3, LAG-3), and establishment of an immunosuppressive stromal barrier via regulatory T cells, myeloid-derived suppressor cells, and cancer-associated fibroblasts. Advanced multiplexed assays combining spatial profiling of immune cell subsets, single-cell RNA sequencing, and proteomic analyses are being developed to capture the multifactorial nature of immune resistance. Ultimately, integrated biomarker panels that interrogate tumor genomics, microenvironmental features, and host immune status hold promise for dynamic patient stratification and adaptive treatment strategies [16].

## Combination strategies:

Combining immunotherapy with other treatment modalities seeks to enhance antitumor efficacy by engaging complementary mechanisms and overcoming intrinsic resistance. One common approach is the addition of immune checkpoint inhibitors to cytotoxic chemotherapy or radiotherapy. Chemotherapy can induce immunogenic cell death, release tumor antigens and increasing dendritic cell uptake, while radiation promotes neoantigen presentation and upregulates adhesion molecules that facilitate T-cell infiltration. Sequential or concurrent scheduling of PD-1/PD-L1 blockade with platinum-based regimens has produced synergistic responses in lung and head and neck cancers, with improvements in overall survival compared to either modality alone [17]. Dual checkpoint blockade targeting two inhibitory receptors simultaneousl leverages nonredundant pathways to amplify T-cell activation. Combinations of CTLA-4 and PD-1 inhibitors have shown higher objective response rates in melanoma and renal cell carcinoma but require careful management of enhanced immune-related toxicities. Emerging combinations target PD-1 alongside LAG-3 or TIGIT, aiming to reinvigorate exhausted T cells that persist despite single-agent therapy. Early clinical data suggest that such regimens can restore effector function in tumors refractory to first-line checkpoint inhibitors. Integrating immunotherapy with targeted agents addresses tumor-driven immunosuppression. For example, VEGF inhibitors normalize abnormal tumor vasculature, reducing hypoxia and facilitating T-cell trafficking, while also modulating regulatory immune populations. Combining anti-angiogenic therapy with PD-1 blockade has produced durable responses in hepatocellular carcinoma and renal cell carcinoma [18]. Similarly, inhibitors of oncogenic kinases (*e.g.*, BRAF/MEK inhibitors in melanoma) can enhance antigen expression and reverse myeloid-derived suppressor cell-mediated suppression, creating an immunopermissive microenvironment. Other innovative combination approaches include pairing immunotherapy with metabolic modulators (such as IDO or adenosine pathway inhibitors) to relieve metabolic checkpoints, and with epigenetic drugs (like DNMT or HDAC inhibitors) to increase tumor immunogenicity. By co-targeting the immune system and tumor cell-intrinsic pathways, these multifaceted strategies aim to convert noninflamed "cold" tumors into inflamed "hot" tumors, thereby broadening the patient population that benefits from immunotherapy [19].

## Safety, toxicity, and management of immune-related adverse events:

Immune-based therapies can provoke a spectrum of off-target inflammatory reactions collectively termed immune-related adverse events (irAEs) that mirror autoimmune disorders and may affect any organ system. The most common irAEs involve the skin (rash, pruritus), gastrointestinal tract (colitis, diarrhea), endocrine glands (thyroiditis, hypophysitis), and liver (hepatitis), although pneumonitis, nephritis, myocarditis, and neurologic toxicities have also been reported [20]. The timing of onset is variable: dermatologic and gastrointestinal events often emerge within weeks of therapy initiation, whereas endocrine irAEs may present months later or even after treatment cessation [21]. Early recognition and grading of irAEs according to established criteria (*e.g.*, CTCAE) are critical to prevent progression to severe or life-threatening complications. Management algorithms recommend holding immunotherapy for grade 2 toxicities and initiating corticosteroids when symptoms fail to improve or escalate to grade 3-4. High-dose steroids remain the cornerstone of treatment for moderate to severe irAEs, with tapering over at least 4-6 weeks to reduce recurrence risk [22]. For steroid-refractory cases, second-line immunomodulators-such as infliximab for colitis, mycophenolate mofetil for hepatitis, or tocilizumab for arthritis can be employed based on organ-specific guidelines. Proactive monitoring protocols, including baseline organ function tests and periodic assessments (*e.g.*, thyroid panels, liver enzymes, pulmonary imaging), enable early detection [23]. Patient education about recognizing warning signs and prompt reporting is essential. Multidisciplinary collaboration with input from gastroenterologists, endocrinologists, pulmonologists, and other specialists is often required for complex or persistent irAEs. Despite the risk of toxicity, most irAEs are reversible with timely intervention, and evidence suggests that transient immunosuppression does not compromise long-term antitumor efficacy [24].

## Challenges and limitations:

Despite transformative clinical successes, several obstacles impede the universal application of immunotherapy across oncology. First, the high cost of novel agents and complex manufacturing processes particularly for personalized cell therapies-poses significant financial burdens for healthcare systems and may limit patient access. Second, tumor heterogeneity, both inter- and intra-patient, leads to variable antigen expression and neoantigen landscapes, reducing the effectiveness of single-target approaches and complicating biomarker development. Third, the immunosuppressive tumor microenvironment characterized by regulatory T cells, myeloid-derived suppressor cells, inhibitory cytokines, and physical barriers like dense stroma can blunt effector T-cell infiltration and function. Overcoming these suppressive networks requires multifaceted strategies but raises concerns about additive toxicity when combining multiple agents. Fourth, mechanisms of primary and acquired resistance, such as loss of antigen presentation machinery, upregulation of alternative checkpoints, and metabolic adaptation by tumor cells, often emerge under selective immune pressure, necessitating ongoing monitoring and adaptive treatment modifications. Finally, managing immune-related adverse events remains a delicate balance between mitigating toxicity and preserving antitumor immunity. The need for robust long-term safety data especially in curative-intent settings and in combination regimens underscores the importance of continued pharmacovigilance. Addressing these challenges will require interdisciplinary collaboration, integration of cutting-edge technologies for tumor and immune profiling, and equitable access frameworks to ensure that the benefits of immunotherapy are realized broadly.

## Future directions and emerging trends:

The next wave of immunotherapeutic innovation is driven by a deeper understanding of tumor-immune dynamics and advances in bioengineering. Novel checkpoint targets beyond PD-1 and CTLA-4 such as LAG-3, TIGIT, and VISTA are entering late-stage clinical evaluation, with bispecific and multifunctional antibody constructs designed to co-engage multiple inhibitory receptors or to couple checkpoint blockade with costimulatory signals. These agents hold promise for reinvigorating T cells that have become refractory to first-line inhibitors. Next-generation cellular therapies are embracing precision engineering to enhance safety, specificity, and persistence. Gene edits that incorporate safety switches or cytokine "armoring" improve control over CAR-T activity and reduce the risk of off-tumor toxicity. Universal ("off-the-shelf") allogeneic CAR-T and CAR-NK products, enabled by CRISPR-mediated disruption of endogenous HLA and TCR loci, aim to overcome logistical barriers and manufacturing delays inherent in autologous approaches. Personalized neoantigen vaccines, informed by tumor exome sequencing and machine-learning algorithms for epitope prediction, are being combined with checkpoint inhibitors to broaden the T-cell repertoire against patient-specific mutations. Simultaneously, oncolytic viral platforms are being armed with transgenes encoding immunostimulatory cytokines or bispecific T-cell engagers, transforming tumors into in situ factories of immune activation. Integration of multimodal biomarker strategies combining single-cell transcriptomics, spatial proteomics, and dynamic liquid-biopsy assays will enable real-time monitoring of immune responses and adaptive treatment tailoring. Artificial intelligence platforms are being trained to predict resistance mechanisms and optimize combination regimens, potentially shortening the path from laboratory discovery to clinical application. Finally, efforts to democratize access to immunotherapy include development of lower-cost vectors, streamlined manufacturing processes, and global clinical networks to evaluate therapies in diverse populations. Together, these emerging trends signal a shift toward truly personalized, precise, and accessible immuno-oncology.

## Conclusion:

Cancer immunotherapy has transformed oncology by enabling durable tumor control through checkpoint blockade and cellular therapies. Continued innovation in biomarkers, combination regimens, and next-generation platforms will be essential to overcome resistance, manage toxicity, and expand patient access.

## References

[1] Zhang Y, Zhang Z (2020). Cell Mol Immunol..

[2] Chow A (2022). Nat Rev Clin Oncol..

[3] Oliveira G, Wu C.J (2023). Nat Rev Cancer..

[4] Liu Y (2023). Mol Cancer..

[5] Bi K (2021). Cancer Cell..

[6] Wei J (2022). Front Immunol..

[7] Huang H (2023). Int J Oncol..

[8] Al Zein M (2023). Drug Discov Today..

[9] Ren X (2021). Annu Rev Immunol..

[10] Bagchi S (2021). Annu Rev Pathol..

[11] Yi M (2023). Mol Cancer..

[12] Li Y (2023). Front Immunol..

[13] Rochere P.D.L (2018). Trends Immunol..

[14] Kaczmarek M (2023). Cells..

[15] Duan L.J (2022). Front Immunol..

[16] Fang P (2022). Front Immunol..

[17] Yu M.W, Quail D.F (2021). Front Immunol..

[18] Tang L (2023). Signal Transduct Target Ther..

[19] Pointer K.B (2022). Trends Cancer..

[20] Huang X (2024). Mol Cancer..

[21] Yao Z (2023). Hum Vaccin Immunother..

[22] Shen J (2022). Front Immunol..

[23] Dong X (2022). Oxid Med Cell Longev..

[24] Mishra A.K (2022). Diseases..

